# Small Bowel Obstruction in Virgin Abdomen: Experience From a Community Center

**DOI:** 10.7759/cureus.101963

**Published:** 2026-01-21

**Authors:** Hania Ahmer, Hamdan Mallick, Amaan Rather, Krishnaraj Mahendraraj, Theodoros Katsichtis

**Affiliations:** 1 Surgery, Bayhealth Hospital, Dover, USA; 2 Surgery, Bayhealth Medical Center, Dover, USA

**Keywords:** adhesive small bowel obstruction, internal abdominal hernia, intestinal obstruction, nonoperative, nonoperative management, virgin abdomen

## Abstract

Background

Small bowel obstruction in patients without prior abdominal surgery, referred to as a “virgin abdomen” (SBO-VA), is historically believed to signal a higher risk of malignancy and therefore mandate exploration. However, recent systematic reviews challenge this dogma, suggesting benign etiologies predominate. This study evaluates the etiology, imaging characteristics, and outcomes of SBO-VA in a modern US cohort.

Methods

A retrospective analysis was performed at Bayhealth Medical Center (2017-2024). Patients with SBO and no prior abdominal surgery were included. Preoperative computed tomography (CT) findings, operative reports, pathology, laboratory values, and postoperative outcomes were analyzed. Given the small sample size, analyses were descriptive and intended to generate hypotheses rather than establish statistical associations.

Results

Fifteen patients were identified; 13 met the inclusion criteria. The mean age was 62 ± 17 years (11 male patients). Abnormal adhesions leading to internal hernia accounted for 38% of cases. Therapeutic exploration occurred in 10 patients (67%), while four patients (31%) had negative exploration. Patients undergoing therapeutic intervention tended to be older and had longer hospital stays compared with those undergoing negative exploration.

CT findings more frequently observed in patients undergoing therapeutic exploration included the presence of a transition point, mesenteric swirl, and stricture or adhesive disease. Hypertension and angiotensin-converting enzyme (ACE) inhibitor use were more commonly observed among patients with negative exploration. Readmissions occurred more frequently in patients with negative exploration. Pathology demonstrated ischemic necrosis in 17% of patients.

Conclusion

SBO-VA in this cohort was predominantly benign, with a low observed rate of malignancy, consistent with contemporary literature. Certain CT features appeared more common in patients requiring therapeutic exploration, while hypertension and ACE inhibitor use were observed more frequently among patients with negative exploration. These findings are descriptive and hypothesis-generating, supporting a selective rather than mandatory approach to exploration in SBO-VA.

## Introduction

Small bowel obstruction (SBO) is a frequent surgical emergency, accounting for a substantial proportion of acute abdominal admissions and up to 50% of emergency laparotomies worldwide [1-3]. Adhesions from prior abdominal surgery remain the most common etiology, present in approximately 80% of cases of SBO [4,5]. However, SBO in a “virgin abdomen” (SBO-VA), defined as an abdomen with no history of prior surgery or abdominal trauma, is an uncommon yet clinically significant scenario [6,7].

In patients with a virgin abdomen, traditional causes such as postoperative adhesions are unlikely. Instead, etiologies may include hernias, tumors, Crohn’s disease, congenital anomalies, gallstone ileus, or other obstructive processes [8-10]. Historically, concerns regarding underlying malignancy have led many centers to advocate for mandatory surgical exploration in these patients [11,12]. However, the evidence supporting this approach is limited and often based on small, older studies [13,14].

Recent literature suggests that most SBO cases in virgin abdomens have benign causes and may be amenable to nonoperative management, potentially avoiding unnecessary laparotomies and their associated morbidity [15-18]. Diagnostic challenges arise due to the lack of prior surgical history, which may delay clinical suspicion or appropriate imaging [19]. Advanced imaging modalities, particularly contrast-enhanced computed tomography (CT), have improved the ability to detect transition points, mesenteric abnormalities, and other predictors of surgical necessity [20-22].

Despite evolving understanding, data regarding incidence, etiology, and outcomes of SBO in the virgin abdomen remain limited. This study aims to characterize SBO in this patient population at a community medical center, focusing on etiology, operative versus nonoperative management, and predictors of therapeutic intervention.

This study aims to descriptively characterize the etiology, imaging findings, management strategies, and short-term outcomes of SBO-VA in a community medical center population.

## Materials and methods

This retrospective observational study was conducted at Bayhealth Medical Center and evaluated adult patients diagnosed with small bowel obstruction (SBO) in the absence of prior abdominal surgery between January 1, 2017, and December 31, 2024. The Bayhealth Institutional Review Board approved the study in December 2024, and informed consent was waived due to the minimal risk and retrospective design.

Consecutive sampling was used, and all eligible patients during the study period were identified through electronic medical record review using Epic SlicerDicer with disease-specific diagnostic and Current Procedural Terminology (CPT) codes. Inclusion criteria consisted of adults aged 18 years and older, no history of abdominal or pelvic surgery, CT-confirmed SBO, and confirmation of the diagnosis by the attending surgeon.

Exclusion criteria included preoperative evidence of incarcerated or strangulated hernias, known inflammatory bowel disease, obstructing malignancy on presentation, palliative or hospice status, and incomplete clinical or imaging data. Demographic variables, clinical features (including fever, tachycardia, abdominal distension, vomiting, obstipation, white blood cell count, and lactate), imaging characteristics (transition point, mesenteric swirl, internal hernia, intussusception, adhesions, and strictures), operative findings, and postoperative outcomes were collected through review of clinical notes, operative reports, radiology images, and pathology results.

Surgeries were categorized as therapeutic, diagnostic non-therapeutic, or negative, and postoperative outcomes included complications, readmissions, recurrence of SBO, and subsequent diagnoses of malignancy or inflammatory bowel disease. Demographic variables, clinical characteristics (including age, comorbidities, prior hernia, baseline inflammatory markers, and severity of presentation), imaging findings, and outcomes were recorded to describe the study population.

Given the small cohort size, analyses were limited to descriptive statistics. Continuous variables are reported as medians with interquartile ranges (IQR), and categorical variables are reported as counts and percentages. Inferential statistical testing was intentionally not performed.

## Results

**Figure 1:**
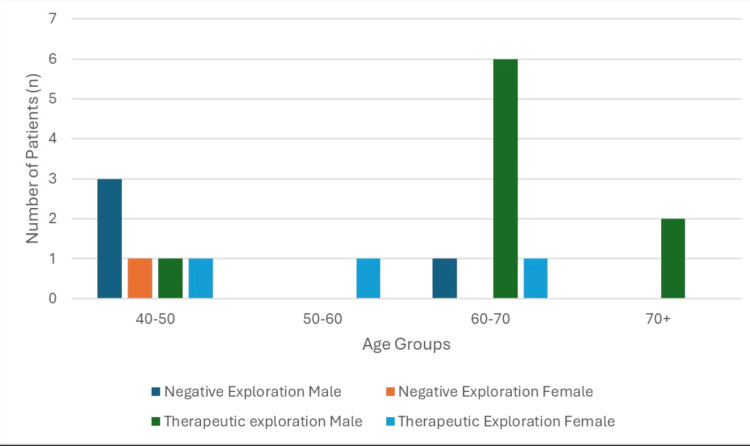
Age group and operative outcome

Fifteen patients were initially identified; two were excluded due to known prior intra-abdominal malignancy, leaving a final cohort of 13 patients. The cohort included 11 men (85%) and two women (15%). The median age was 63 years (IQR: 49-74). Ten patients (77%) underwent operative exploration, while three patients (23%) were managed nonoperatively. Older patients more frequently underwent therapeutic intervention, whereas younger patients more often had negative exploration (Figure 1).

Five patients (38%) had abnormal adhesions resulting in internal hernia formation. Negative exploration occurred in four patients (31%) with a median follow-up of four weeks. Two patients (15%) had isolated strictured segments of small bowel, with benign pathology and no evidence of Crohn’s disease. One patient with intermittent dilation and collapse of small bowel loops is undergoing evaluation for inflammatory bowel disease. Two patients with negative exploration were readmitted for partial SBO and managed conservatively.

The median hospital length of stay was five days (IQR: 4-7). Two patients with negative exploration were readmitted with recurrent partial SBO managed conservatively.

Hypertension was present in eight patients (62%). Among hypertensive patients, five were taking angiotensin-converting enzyme (ACE) inhibitors. Negative exploration occurred in three of the eight hypertensive patients. Other comorbidities included diabetes mellitus (four patients), atrial fibrillation (three patients), congestive heart failure (two patients), and end-stage renal disease (one patient) (Table 1).

**Table 1:** Patient comorbidities by operative outcome ACE: angiotensin-converting enzyme

Comorbidity	Total cohort (N=13)	Negative exploration (n=4)	Therapeutic exploration (n=9)
Hypertension	8 (62%)	3 (75%)	5 (56%)
Diabetes mellitus	4 (31%)	0 (0%)	4 (44%)
Atrial fibrillation	3 (23%)	0 (0%)	3 (33%)
Congestive heart failure	2 (15%)	0 (0%)	2 (22%)
End-stage renal disease	1 (8%)	0 (0%)	1 (11%)
ACE inhibitor use	5 (38%)	2 (50%)	3 (33%)

Preoperative CT findings such as transition points, mesenteric swirl, and adhesion or stricture were more frequently observed in patients who underwent therapeutic exploration, whereas internal hernia and intussusception were not (Table 2).

**Table 2:** Preoperative CT imaging findings CT: computed tomography

CT finding	Total cohort (N=13)	Negative exploration (n=4)	Therapeutic exploration (n=9)
Transition point	11 (85%)	3 (75%)	8 (89%)
Mesenteric swirl	1 (8%)	0 (0%)	1 (11%)
Adhesion/stricture suspected	2 (15%)	0 (0%)	2 (22%)
Internal hernia	1 (8%)	0 (0%)	1 (11%)
Intussusception	1 (8%)	0 (0%)	1 (11%)

Readmissions were more frequently observed among patients who underwent negative exploration (Table 3).

**Table 3:** Postoperative outcomes SBO: small bowel obstruction

Outcome	Total cohort (N=13)	Negative exploration (n=4)	Therapeutic exploration (n=9)
Postoperative ileus	4 (31%)	1 (25%)	3 (33%)
Readmission for SBO	2 (15%)	2 (50%)	0 (0%)
In-hospital mortality	1 (8%)	1 (25%)	0 (0%)

Pathology demonstrated ischemic necrosis in one patient (8%), transmural inflammation in one patient (8%), and normal bowel tissue in two patients (15%). No obstructing malignancies were identified.

## Discussion

Historically, SBO-VA was managed with mandatory operative exploration due to concern for occult malignancy [11-14]. In contrast, our cohort demonstrated predominantly benign etiologies, consistent with contemporary systematic reviews [15-18,21-23].

It is important to note that patients with known obstructing malignancy were excluded by study design, which likely contributed to the low malignancy rate observed. Therefore, these findings should not be interpreted as representative of malignancy prevalence in all SBO-VA populations.

Negative exploration occurred in nearly one-third of patients, highlighting diagnostic challenges in this population. Hypertension and ACE inhibitor use were more commonly observed among patients with negative exploration; however, this observation should be interpreted cautiously, given the small sample size and inability to perform multivariable analysis. Prior reports have described ACE inhibitor-associated bowel angioedema as a potential mimicker of obstruction [18,19]. In addition, hypertension itself has been associated with chronic mesenteric microvascular changes and impaired autoregulation, which may contribute to transient bowel wall edema or dysmotility, potentially increasing the likelihood of negative exploration. Although causation cannot be determined, our findings suggest a need for heightened awareness of these comorbidities when evaluating and stratifying patients with SBO-VA.

Our study further reinforces the critical role of CT imaging in the modern management of SBO-VA. Prior work has demonstrated that CT reliably identifies transition points, strangulation signs, mesenteric edema, and other markers predictive of the need for surgery [20-22].

Our results also demonstrate that nonoperative management is often successful, consistent with emerging evidence challenging the historic mandatory laparotomy doctrine. Multiple contemporary studies, including large analyses from the ANZ Journal of Surgery and the Journal of the American College of Surgeons (JACS), have concluded that selective nonoperative management is both safe and effective in a majority of patients with SBO-VA [15-18,21-23]. Our data support this practice, as several patients with negative explorations or partial obstructions ultimately recovered with conservative therapy. However, two patients who initially underwent negative exploration experienced recurrent SBO episodes managed nonoperatively, highlighting the importance of follow-up and consideration of alternative underlying diagnoses such as intermittent volvulus, early inflammatory bowel disease, or medication-induced enteropathy.

Limitations include the study’s retrospective design, small sample size, and short follow-up duration. These factors restrict generalizability and prevent robust multivariable analysis. Nonetheless, our findings contribute contemporary, real-world data and strengthen the argument for tailored, evidence-based decision-making in SBO-VA.

## Conclusions

Small bowel obstruction in the virgin abdomen was most commonly associated with benign pathology in this community cohort. A substantial proportion of patients experienced negative surgical exploration. Individualized management guided by clinical assessment and CT imaging may help reduce unnecessary surgery. Larger prospective and multicenter studies are warranted to validate these findings and refine management algorithms.
